# Comparative Effects of At-Home and In-Office Bleaching on Discoloration Due to Treatment of White Spot Lesions With Silver Diamine Fluoride

**DOI:** 10.1155/ijod/6837096

**Published:** 2025-10-05

**Authors:** Farzaneh Shirani, Leili Mirmiran, Abolfazl Mirmiran

**Affiliations:** ^1^Department of Restorative Dentistry, Dental Materials Research Center, Dental Research Institute, School of Dentistry, Isfahan University of Medical Sciences, Isfahan, Iran; ^2^School of Dentistry, Isfahan University of Medical Sciences, Isfahan, Iran

**Keywords:** dental caries, fluorides, silver diamine fluoride, tooth bleaching, tooth discoloration

## Abstract

**Objectives:**

Incipient enamel caries, known as white spot lesions (WSLs), can be prevented or halted with proper oral hygiene, diet control, and application of remineralizing agents such as silver diamine fluoride (SDF); however, dark staining is a major drawback of SDF. This study aimed to compare the effects of at-home and in-office bleaching on SDF-induced staining of WSLs.

**Materials and Methods:**

In this in vitro study, 44 bovine central incisors were randomly assigned to 2 groups (*n* = 22) for artificial induction of superficial and deep WSLs. Next, both groups underwent remineralization by using 38% SDF for 1 min, rinsed, and stored in artificial saliva. After 1 week, each group was randomly divided into two subgroups (*n* = 11) to undergo at-home bleaching and in-office bleaching. The *L*^*⁣*^*∗*^^, *a*^*⁣*^*∗*^^, and *b*^*⁣*^*∗*^^ color parameters of all specimens were measured at 4 time points of baseline (sound enamel), after demineralization, after SDF remineralization, and after bleaching, and the color change (Δ*E*_00_) was calculated. Data were analyzed by one-way and two-way ANOVA and Sidak-adjusted post hoc test (*α* = 0.05).

**Results:**

Lesion depth did not significantly affect early-stage discoloration (*p*  > 0.05). However, a significant interaction was found between lesion depth and bleaching type at later stages (*p*  < 0.001). In-office bleaching significantly improved Δ*E*_00_ compared to at-home bleaching but only in superficial lesions (*p*  < 0.001).

**Conclusion:**

Both at-home and in-office bleaching can lighten dark lesions caused by SDF after remineralization, although they may not fully restore tooth color to normal. In-office bleaching led to significantly greater color improvement than at-home bleaching in superficial lesions.

## 1. Introduction

The efficacy of fluoride for reducing the incidence of dental caries is approaching its threshold at the community level, and researchers are trying to develop new methods for enamel remineralization [1]. Silver diamine fluoride (SDF) is a clear liquid that possesses the antibacterial effects of silver and the remineralizing properties of fluoride. It is used to manage carious lesions in children and people requiring special care [2]. SDF is effective as a desensitizer and a cariostatic agent. Silver and fluoride play important roles in preventing caries and treating teeth with dentin hypersensitivity. By using SDF, the caries progression process can be halted, and the state of carious lesions can be changed from acute to chronic, even in cavitated lesions [2]. However, the main drawback of SDF is its tendency to darken porous enamel and carious dentin due to silver deposition [3], causing SDF-induced staining, which is unacceptable to patients, especially in the esthetic zone [4]. Recent studies have explored several alternative strategies to address this issue, such as replacing silver with nanosilver particles, using glutathione or potassium iodide, and applying glass ionomer cement after utilizing potassium iodide [5–7]. However, no consensus has been reached in this regard.

Dental bleaching is a safe and effective method for the correction of tooth discoloration. Its mechanism of action is based on the oxidation of organic pigments in teeth [8]. Hydrogen peroxide (H_2_O_2_) and carbamide peroxide are the active substances used in most bleaching protocols [9]. Power (in-office) bleaching and at-home bleaching are the two commonly used bleaching protocols. In at-home bleaching, patients fill a special tray with the bleaching agent and place it in their mouth for several hours according to the provided instructions. In the in-office bleaching method, a high concentration of H_2_O_2_ is used for a shorter period of time under the supervision of a dental clinician [10].

Al-Angari et al. [11] investigated the effect of bleaching on remineralized artificial caries and found that artificial carious lesions that had been stained with nonmetallic compounds, such as colored foods, during remineralization, were more responsive to bleaching treatment than lesions stained with metallic compounds, such as SDF. However, de Geus et al. [12] evaluated the difference between at-home bleaching and in-office bleaching, and found no significant difference between them regarding the risk or intensity of tooth hypersensitivity or the effectiveness of bleaching treatment.

Restorative or cosmetic treatments of teeth with SDF-induced discoloration should be done with minimal removal of the tooth structure. SDF can be used in superficial lesions, mainly when the goal is caries arrest in patients who cannot tolerate restorative care immediately, or as a preventive/temporary measure in high-risk patients [13]. Therefore, the present study was carried out to compare the effects of at-home and in-office bleaching protocols on SDF-induced discoloration of superficial and deep artificially induced white spot lesions (WSLs).

## 2. Materials and Methods

**Figure 1:**
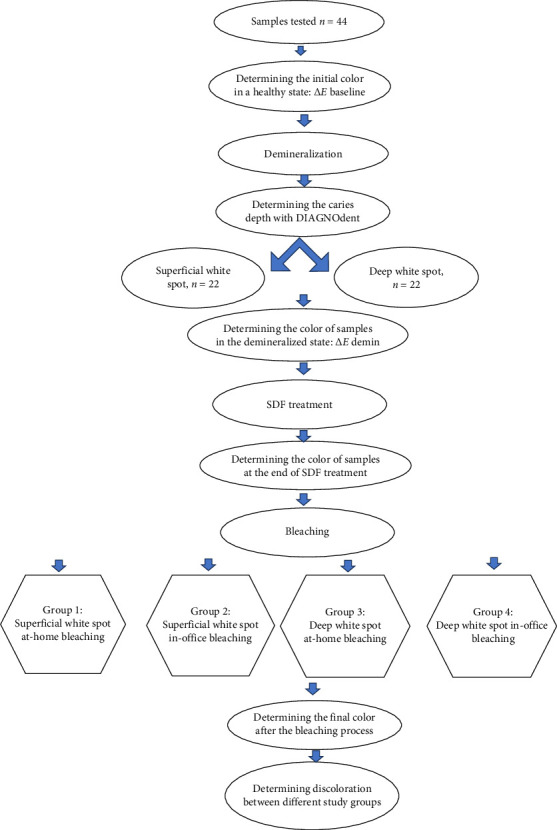
Flowchart of the procedural steps of the study.

This in vitro, experimental study was conducted on 44 extracted bovine mandibular anterior teeth collected within 6 months. The included teeth were sound and had no discoloration, caries, wear, fracture, or hypoplastic lesions. This study protocol was ethically approved by the ethics committee of the university (IR.MUI.RESEARCH.REC.1400.255). Although the study had an in vitro design, complete compliance with local ethical regulations regarding animal tissues was ensured.  Figure 1 shows the procedural steps of the study.

### 2.1. Sample Size

A priori power analysis was conducted using G^*⁣*^*∗*^^Power version 3.1 to determine the minimum required sample size. The study design included two predictors (lesion depth and bleaching type), two dependent variables (Δ*E*_00_ from sound enamel to postbleaching, and from post-SDF to postbleaching), and four groups in total. An effect size of *f*^2^(*V*) = 0.15 (a medium effect size according to Cohen's guidelines) was used. With the significance level set at *α* = 0.05 and desired power at 0.80, the analysis indicated that at least 43 participants were needed. To enhance the robustness of the study and allow for potential variability or data loss, the sample size was increased to 48 (12 participants per group) [14].

### 2.2. Specimen Preparation

The collected teeth were cleaned from soft tissue residues with a scalpel and polishing brush, immersed in 0.2% thymol solution for disinfection, rinsed with water, and stored in artificial saliva at 37°C with a pH of 7 for simulation of the oral environment. The composition of artificial saliva included 20 mmol/L NaHCO_3_, 3 mmol/L NaH_2_PO_4_, and 1 mmol/L CaCl_2_, and it was refreshed on a daily basis.

The teeth were then decoronated at the cementoenamel junction by a cutting machine (Vafaei Industrial CO, Tehran, Iran) under water coolant. Prior to color measurement, all teeth were cleaned with a low-speed hand-piece and a combination of fluoride-free and oil-free pumice paste and water. They were then rinsed with water. Each tooth was then mounted in a putty mold (Sil Set, Hanan Shimi, Iran) to standardize the tooth position for repeated color measurements. The tip of the spectrophotometer (Shade Pilot; DeguDent, Germany) was fixed on the tooth surface in a reproducible position. The mold dimensions matched the size of the Shade Pilot tip. The part of the tooth surface subjected to color measurement was marked with a white sticker measuring 4 mm× 4 mm. The remaining tooth surface was coated with acid-resistant black nail varnish [15]. All teeth underwent baseline color assessment using a Shade Pilot spectrophotometer.

### 2.3. Demineralization of Specimens

Each tooth was then placed in a coded test tube containing 5 mL of demineralizing solution composed of 2.2 mmol/L KH_2_PO_4_, 2.2 mmol/L CaCl_2_, and 50 mmol/L acetic acid at a pH of 5 for 24 h to artificially induce incipient enamel lesions. The solution in each test tube was changed every day until a chalky white appearance was achieved on the tooth surface. The extent of the WSL on each tooth was checked every day with a DIAGNOdent (Kavo Dental GmbH, Germany) until the desired depth of demineralization was achieved. The time required for development of a WSL on the outer surface of enamel with a depth half the enamel depth was 10–25 days, and the time required for development of a deep enamel lesion was 26–37 days [15]. Based on the numbers shown by the DIAGNOdent, the teeth were divided into two groups with superficial and deep incipient enamel lesions. The DIAGNOdent values range from 0 to 10 for sound tooth structure, 11–20 for outer-half enamel caries, 21–30 for inner-half enamel caries, and +30 for dentin caries [15]. The color of WSLs was measured in each group using the Shade Pilot spectrophotometer. After the induction of superficial and deep WSLs, each group was randomly assigned to two subgroups (*n* = 11) for in-office and at-home bleaching. All groups were then stored in artificial saliva.

### 2.4. Remineralization of Specimens

Next, 38% SDF (Advantage Arrest, USA) was applied on the artificially induced superficial and deep WSLs for 1 min. After waiting for 1 min, the remaining moisture was dried with a tissue. The specimens were placed in artificial saliva at 37°C for 1 week. After 1 week of incubation, the WSLs on the teeth showed a black discoloration.

### 2.5. Bleaching Procedures

**Figure 2:**
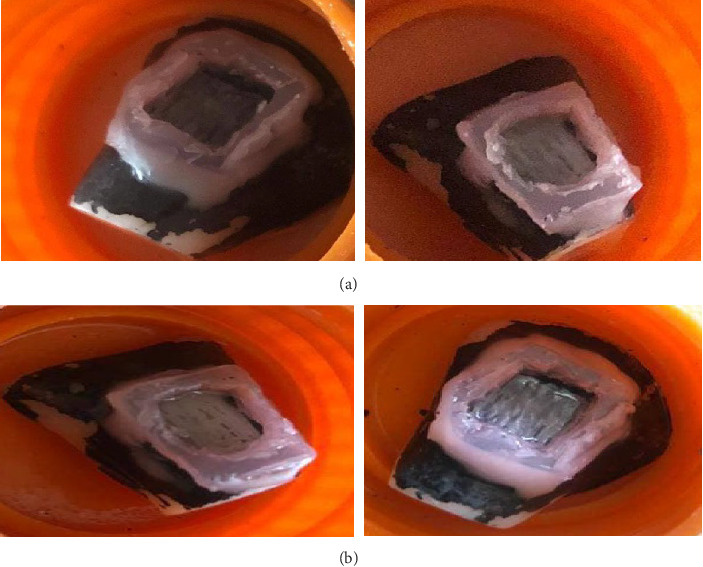
(A) Specimens before bleaching and (B) the same specimens 1 week after bleaching.

Groups 1 and 3 were treated with 15% carbamide peroxide at-home bleaching agent (Opalescence Boost, Ultradent Products, Inc., South Jordan, UT, USA). A 0.5–1 mm layer of bleaching gel was applied on the enamel stain resulting from SDF treatment, and incubated for 6 h a day at 37°C for 7 days. After bleaching, the specimens were rinsed with distilled water for 1 min to remove the bleaching gel. They were then dried with paper towels and stored in a humidified environment at 4°C. Groups 2 and 4 were treated with 35% H_2_O_2_ in-office bleaching agent (White Smile 2019, Germany). A 0.5–1 mm layer of bleaching agent was applied on the enamel and dentin stain resulting from SDF treatment. After 60 min, the bleaching gel was removed with a cotton roll. This treatment was repeated 6 more times in subsequent sessions. The specimens were then washed with distilled water for 1 min, dried with a paper towel, and stored in a humid environment at 4˚C until their color measurement [11] (Figure 2).

### 2.6. Assessment of Color Change (Δ*E*_00_)

The color parameters of each specimen were recorded using the CIE *L*^*⁣*^*∗*^^*a*^*⁣*^*∗*^^*b*^*⁣*^*∗*^^ color space, with *L*^*∗*^ indicating lightness, *a*^*∗*^ indicating greenness-redness, and *b*^*∗*^ indicating blueness-yellowness. These values were converted to the CIEDE2000 color parameters. The color difference between time periods (Δ*E*_00_) was calculated using the CIEDE2000 formula as follows [16]:

  $$\begin{array}{c} \Delta E_{00}=\sqrt{{\left(\frac{\Delta L^{\prime }}{K_{L}S_{L}}\right)}^{2}+{\left(\frac{\Delta C^{\prime }}{K_{C}S_{C}}\right)}^{2}+{\left(\frac{\Delta H^{\prime }}{K_{H}S_{H}}\right)}^{2}+R_{T}\left(\frac{\Delta C^{\prime }}{K_{C}S_{C}}\right)\left(\frac{\Delta H^{\prime }}{K_{H}S_{H}}\right)}, \end{array}$$

where Δ*L*′, Δ*C*′, and Δ*H*′ represent the differences in lightness, chroma, and hue, respectively. The weighting functions *S*_*L*_, *S*_*C*_, and *S*_*H*_ adjust the influence of each component, while *K*_*L*_, *K*_*C*_, and *K*_*H*_ are parametric correction factors. *R*_*T*_ represents a rotation function that accounts for the interactive effect between chroma and hue differences in the blue region of the color space.

### 2.7. Statistical Analysis

Statistical analyses were performed using SPSS version 26 (IBM Corp., Armonk, NY, USA), and bar charts were generated with GraphPad Prism version 9.0 (GraphPad Software, San Diego, CA, USA). To investigate how lesion depth influenced early discoloration (Δ*E*_00_ from sound enamel to demineralized and post-SDF stages), a one-way MANOVA was applied with two dependent variables. For the later stages involving bleaching, a two-way MANOVA was used to examine the effects of both lesion depth and bleaching type on Δ*E*_00_ (from sound enamel to postbleaching, and from post-SDF to postbleaching). When significant interactions were detected, Sidak-adjusted post hoc comparisons were conducted. All assumptions of multivariate analysis, including normality, homogeneity of variances, and equality of covariances, were checked and met. The significance level was set at *α* = 0.05.

## 3. Results

The DIAGNOdent values of the specimens are presented in Table 1.

### 3.1. Early Discoloration

Descriptive statistics for the Δ*E*_00_ of the first two stages are shown in Table 2. One-way MANOVA was applied to analyze the effect of lesion depth (superficial vs. deep) on Δ*E*_00_ from sound enamel to demineralized, and from sound enamel to post-SDF. The analysis showed no significant multivariate effect of lesion depth with Wilks' lambda = 0.987, *F* (2, 45) = 0.303, *p* = 0.740, and partial *η*^2^ = 0.013 (Table 3). Univariate ANOVA also confirmed that differences among the groups were not statistically significant for either Δ*E*_00_ value (demineralization: *F* [1, 46] = 0.12, *p* = 0.728; post-SDF: *F* [1, 46] = 0.53, and *p* = 0.473) (Table 4).

### 3.2. Bleaching Stages

Descriptive statistics for the bleaching stages are presented in Table 5. Two-way MANOVA was applied to analyze the effects of bleaching type (home vs. office) and lesion depth on two dependent variables: Δ*E*_00_ from sound enamel to postbleaching, and from post-SDF to postbleaching. The analysis revealed a statistically significant interaction between lesion depth and bleaching type with Wilks' lambda = 0.723, *F* (2, 43) = 8.252, *p* = 0.001, partial *η*^2^ = 0.277 (Table 6). This interaction was significant only for the Δ*E*_00_ from post-SDF to postbleaching (*F* [1, 46] = 14.87, *p*  < 0.001); while the Δ*E*_00_ from sound enamel to postbleaching showed no significant interaction (*F* [1, 46] = 0.075, *p* = 0.785) (Table 7).

**Figure 3:**
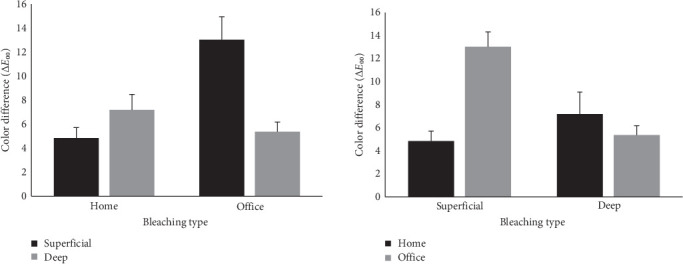
Interaction effects of lesion depth and bleaching type on Δ*E*_00_ from post-SDF to postbleaching. Significant differences were determined using Sidak-adjusted post hoc tests. Asterisks indicate significant differences (*⁣*^*∗*^*p*  < 0.001); “ns” indicates nonsignificant comparisons.

Given the significant interaction, main effects were not interpreted independently. Instead, Sidak-adjusted pairwise comparisons were conducted to further explore the interaction, and the results are visualized in Figure 3.

As illustrated in Figure 3, pairwise comparisons showed that in-office bleaching led to significantly greater color improvement than at-home bleaching, but only in superficial lesions (*p*  < 0.001). In deep lesions, no significant difference was found between bleaching types (*p* = 0.327). Additionally, within the in-office bleaching group, superficial lesions exhibited significantly more color change than deep lesions (*p*  < 0.001); whereas, this difference was not significant in the at-home bleaching group (*p* = 0.209).

## 4. Discussion

One suggested strategy to mask the SDF-induced discoloration is to remove part of the discolored tooth structure and restore it with tooth-colored restorative materials. In this study, an attempt was made to preserve the tooth structure and restore the form, function, and esthetic appearance of carious teeth treated with SDF because it has been proven that preserving the tooth structure as much as possible and performing fewer invasive interventions can strongly improve the clinical durability of teeth [14].

This study compared the effects of at-home and in-office bleaching protocols on SDF-induced discoloration of superficial and deep artificially induced WSLs. The visual results indicated that the whiteness of the teeth was higher after the application of SDF in deep caries than in superficial caries. One reason for this difference may be that deep lesions have greater porosities, providing more space for penetration of SDF and binding of silver to tooth structure. Therefore, SDF is spread in a larger area because a specific volume of SDF is applied to the lesion in one application, and less darkening is observed on the lesion surface. The present results showed that in-office bleaching led to significantly greater color improvement than home bleaching, but only in superficial lesions. In deep lesions, no significant difference was found between the two bleaching types.

One previous study showed that in-office bleaching agents provided higher Δ*E* values than at-home bleaching agents and had a greater effect on restoring the lightness of the teeth [17]. However, another study showed that both at-home and in-office bleaching methods had similar effects on discolorations caused by compounds other than SDF [12]. Considering the greater power of bleaching agents used for in-office bleaching, this method appears to be more suitable for the correction of SDF-induced discoloration because this discoloration is due to the chemical interaction of silver with tooth structure and requires a highly potent bleaching agent [18].

Bleaching is an effective strategy to correct SDF-induced discoloration of carious teeth remineralized with SDF since it preserves the tooth structure [1]. In this study, at-home and in-office bleaching methods were chosen to compare their specific advantages and disadvantages for this purpose. The in-office method with greater bleaching power and the at-home method with higher reproducibility can be used to correct SDF-induced discoloration [19].

The reason for the lack of a statistical difference in efficacy of in-office and at-home bleaching for deep WSLs in the present study can be the limited sample size. The Δ*E*_00_ between the sound and demineralized enamel showed that the teeth became visually lighter, which was due to the formation of porosities in the body of WSLs and further reflection of light from this part of the tooth compared to sound enamel.

In comparison of sound and SDF-remineralized enamel, due to the deposition and interaction of silver with the tooth structure, a large Δ*E*_00_ was noted. SDF forms an insoluble protective layer by forming insoluble or hardly soluble compounds, including calcium fluoride, silver phosphate, and silver protein complexes, and their deposition on the tooth surface, leading to a reduction in demineralization.

In comparison of sound and bleached enamel, a high Δ*E*_00_ was still observed, indicating a color difference between the sound and bleached enamel, but this value was largely lower compared to SDF-remineralized enamel, indicating a visually lighter color [14] (Figure 2). This result is promising for more conservative treatments of tooth discoloration following SDF application to control acute caries.

Bovine anterior teeth were used in this study due to their larger size, which simplifies experimental procedures. While there are some differences between bovine and human teeth, as in their anatomical structure, bovine and human teeth share key chemical and physical properties, including similar enamel composition, number of dentinal tubules, and microhardness [20–22]. However, it is important to recognize that bovine teeth may not fully replicate the behavior of human teeth in the clinical setting; therefore, generalization of the results to human teeth should be done with caution.

While bleaching agents primarily target organic compounds in the tooth structure and have a limited effect on metallic discolorations, chemical agents such as nitric acid may offer a promising alternative. Nitric acid can dissolve the silver-tooth phosphate complex formed by SDF, leading to a permanent color change. This approach may provide a solution for eliminating SDF-induced discoloration, offering a potentially novel treatment strategy. However, further investigations, including studies on the biocompatibility and safety of these chemical agents, are essential before their integration into clinical practice for managing esthetic concerns associated with SDF treatment.

## 5. Conclusion

Both at-home and in-office bleaching can lighten dark lesions caused by SDF after remineralization, although they may not fully restore tooth color to normal. In-office bleaching led to significantly greater color improvement than at-home bleaching in superficial lesions.

### 5.1. Ethical Considerations and Limitations

This study was ethically approved by the ethics committee of the university (IR.MUI.RESEARCH.REC.1400.255). Although the study had an in vitro design, complete compliance with local ethical regulations regarding animal tissues was ensured.

This study had some limitations. The depth of WSLs was only measured by DIAGNOdent, while it would be ideal to histologically determine the depth of lesions. Also, the at-home bleaching protocol may not reflect standard patient compliance or real-world practice. Use of bovine teeth instead of human teeth and in vitro design were among other limitations that limit the generalizability of the findings. Future studies are recommended to address the abovementioned limitations and obtain more accurate results with higher generalizability.

## Figures and Tables

**Table 1:** DIAGNOdent values of the specimens.

Specimen number	Mean DIAGNOdent value for superficial WSLs	Specimen number	Mean DIAGNOdent value for deep WSLs
2	14	1	43
3	14	4	20
5	12	10	20
6	11	11	23
7	14	12	20
8	12	14	32
9	13	15	21
13	15	16	31
17	14	20	28
18	12	21	23
19	14	22	29
23	13	24	21
30	11	25	20
31	16	26	40
32	12	27	21
34	14	28	22
35	14	29	23
38	11	33	21
40	14	36	20
41	13	37	22
42	15	39	26
43	13	44	24

**Table 2:** Mean and standard deviations of Δ*E*_00_ after demineralization and SDF application by lesion depth.

Lesion depth	Δ*E*_00_ (sound → demineralized)	Δ*E*_00_ (sound → post-SDF)
Superficial	6.31 ± 2.13	38.24 ± 10.92
Deep	6.55 ± 2.62	40.54 ± 11.03
Total	6.43 ± 2.36	39.39 ± 10.92

*Note*: Values represent mean ± standard deviation of Δ*E*₀₀ based on the CIEDE2000 formula.

**Table 3:** MANOVA for the effect of lesion depth on Δ*E*_00_ in the first two stages (Δ*E*_00_: sound → demineralized, sound → post-SDF).

Effect	Wilks' lambda	*F* (*df*)	*p*-Value	Partial *η*^2^	Interpretation
Lesion depth	0.987	*F* (2, 45) = 0.303	0.740	0.013	Not significant

**Table 4:** Univariate ANOVA results for Δ*E*_00_ in the first two stages (Δ*E*_00_: sound → demineralized, sound → post-SDF).

Stage	*F* (1, 46)	*p*-Value	Partial *η*^2^	Interpretation
Sound → demineralized	0.122	0.728	0.003	Not significant
Sound → post-SDF	0.525	0.473	0.011	Not significant

**Table 5:** Mean and standard deviation for later stages of Δ*E*_00_ by lesion depth and bleaching type.

Lesion depth	Bleaching type	Δ*E*_00_: sound → postbleaching	Δ*E*_00_: post-SDF → postbleaching
Superficial	Home	30.97 ± 7.25	4.85 ± 3.01
	Office	29.47 ± 13.61	13.03 ± 6.63

Deep	Home	36.64 ± 10.69	7.19 ± 4.45
	Office	33.57 ± 6.22	5.37 ± 2.81

*Note*: Values represent mean ± standard deviation of Δ*E*₀₀ based on the CIEDE2000 formula.

**Table 6:** MANOVA for the effects of lesion depth and bleaching type on later stages of color change (Δ*E*_00_: sound → postbleaching, SDF → postbleaching).

Effect	Wilks' lambda	*F* (*df*)	*p*-Value	Partial *η*^2^	Interpretation
Lesion depth	0.887	*F* (2, 43) = 2.739	0.076	0.113	Marginal (not significant)
Bleaching type	0.879	*F* (2, 43) = 2.951	0.063	0.121	Marginal (not significant)
Lesion depth × bleaching type	0.723	*F* (2, 43) = 8.252	0.001	0.277	Significant

**Table 7:** Univariate ANOVA results for Δ*E*_00_ in later stages (Δ*E*_00_: sound → postbleaching and post-SDF → postbleaching).

Stage	Source	*F* (1, 46)	*p*-Value	Partial *η*^2^	Interpretation
Sound → postbleaching	Lesion depth	2.93	0.094	0.063	Marginal (not significant)
	Bleaching type	0.64	0.428	0.014	Not significant
	Lesion depth × bleaching type	0.075	0.785	0.002	Not significant

SDF → postbleaching	Lesion depth	4.21	0.046	0.087	Significant
	Bleaching type	6.03	0.018	0.120	Significant
	Lesion depth × bleaching type	14.87	<0.001	0.253	Significant interaction

## Data Availability

The data used to support the findings of this study were supplied by the corresponding author under license, and the data will be available upon request. Requests for access to these data should be made to the corresponding author.
